# Heat generation and histological analysis of peri-implant bone cutting using piezoelectric surgery for implant removal: an in vitro study

**DOI:** 10.1186/s40729-025-00658-y

**Published:** 2025-12-29

**Authors:** Yoshiki Saito, Hiroki Tsukamoto, Kiyoshi Shimada, Yusuke Kondo, Keiko Kiyomura, Tomotaka Nodai, Takashi Munemasa, Taro Mukaibo, Ryuji Hosokawa, Chihiro Masaki

**Affiliations:** Division of Oral Reconstruction and Prosthodontics, Kyushu Dental University, 2-6-1, Manazuru, Kokurakita-Ku, Kitakyushu, Fukuoka 803-8580 Japan

**Keywords:** Dental implant, Piezoelectric surgery, Implant removal, Temperature increase, Thermal injury

## Abstract

**Purpose:**

This study aimed to evaluate the safety of piezoelectric surgery for bone cutting during implant removal in terms of heat generation and histological changes.

**Methods:**

The bone model experiments involved titanium implants or non-metal dummy implants placed in bone models. Bone cutting using a piezoelectric surgery with irrigation was performed at distances of 0, 1, and 2 mm from the implant, and the temperature was recorded using a contact thermometer placed at the tip of the implant. Using procine mandible models, histological analysis was performed using hematoxylin and eosin-stained images to evaluate the risk of thermal injury.

**Results:**

When bone cutting was performed at distances of 0 mm from the implants continuously, a significantly greater temperature increase was observed with the titanium implants compared to the non-metal dummy implants. The increase in temperature decreased as the distance between the implant and the cutting position increased. Comparing the cutting patterns, a greater temperature increase was observed with continuous and 10 s intermittent cutting. In contrast, it was suppressed with intermittent cutting for 3 and 5 s. In the histological analysis with porcine mandibles, findings suggest that thermal injury was not observed in any of the samples.

**Conclusions:**

No bone damage was observed in the histological analysis. In contrast, piezoelectric peri-implant bone cutting caused a significant increase in temperature, especially for continuous bone cutting during implant adjustments. However, intermittent cutting for 3 and 5 s significantly suppressed the temperature increase. The results suggest that shortening the continuous cutting time may be effective in preventing heat generation when using piezoelectric surgery for peri-implant bone cutting.

## Background

Dental implants, based on the osseointegration of titanium and bone, has become an essential treatment option for patients with missing teeth, offering both functionality and predictability [1, 2]. The number of patients undergoing implant treatments has continued to increase annually [3, 4]. With the increasing number of implants placed, the frequency of implant removal due to peri-implantitis, implant fractures, or positional discrepancies has also elevated. In patients requiring implant replacement in the same site, implant removal techniques that are less traumatic and preserve as much of the surrounding bone as possible are critically important. Currently, the reverse torque technique, which involves reversing the implant’s rotation using dedicated instruments, is considered the most effective approach [5]. Using this technique, the success rate is high [6, 7]. Nevertheless, it is difficult to apply the reverse torque technique in cases with strong osseointegration, incompatible implant designs, or implant fractures. In these situations, peri-implant bone cutting is unavoidable. Although conventional methods using rotary instruments, such as trephine burs, are effective, they are also associated with significant drawbacks, including circumferential bone cutting, excessive bone loss, and an increased risk of bone damage [8].

Piezoelectric surgery has been widely adopted in oral and maxillofacial surgery, such as orthognathic surgery, maxillary sinus elevation, bone graft harvesting, and implant site preparation, because of its minimal impact on the soft tissues [9–12]. Piezoelectric surgery may also be useful when peri-implant bone cutting is required for implant removal. However, a standardized protocol for safe and effective implant removal using piezoelectric surgery has not yet been established. In particular, cutting narrow and deep bone sites, where adequate irrigation may be difficult, may lead to heat accumulation and subsequent thermal injury. Previous studies, such as the study by Lamazza et al., have shown that insufficient irrigation increases the temperature at the site [13]. A systematic review comparing heat generation during bone drilling for implant placement using conventional rotary instruments and piezoelectric surgery has also been published [14]. While the variability in temperature was not the focus of the review, many of the included studies indicated that piezoelectric surgery tends to generate higher temperatures [15–19]. More importantly, a greater temperature increase when cutting close to implants using piezoelectric surgery may cause higher heat generation as a result of thermal conversion of vibration energy when contacting metal. To date, no studies have investigated this.

This in vitro study aimed to evaluate the safety of piezoelectric surgery for bone cutting during implant removal in terms of heat generation and histological changes.

## Methods

### Evaluation of temperature increase in a bone model

**Fig. 1:**
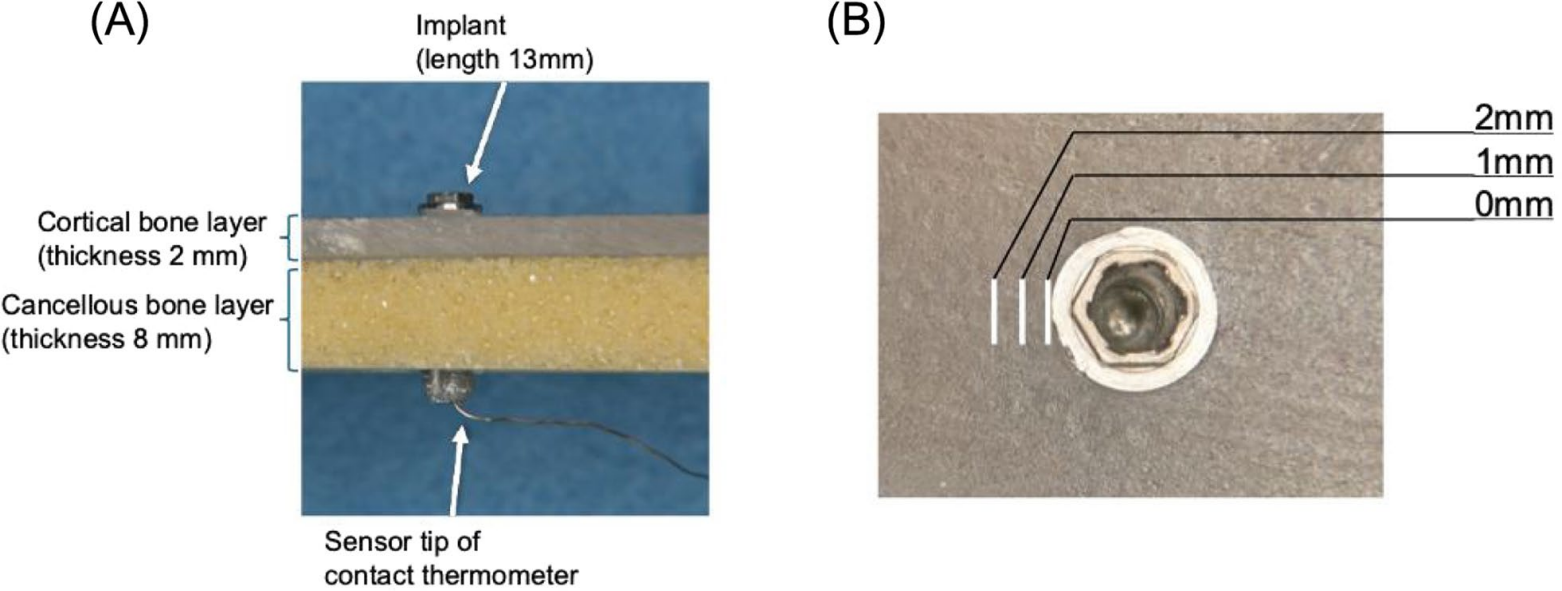
Schematic illustration of the experimental setup. **A** Implant placement into the bone model and temperature measurement at the implant apex. **B** Position of the piezoelectric surgery blade during bone cutting. Cutting was performed at positions of 0 mm (adjacent to the implant), 1 mm, and 2 mm away from the implant, respectively

The titanium implants (diameter: 4.0 mm, length: 13 mm; Brånemark System® MkⅣ, Nobel Biocare, Zürich, Switzerland) or non-metal dummy implants, which were fabricated using a chemically cured composite resin (Protemp™ 4, 3 M Deutschland GmbH, Neuss, Germany), were placed into the layered bone model (Sawbones®, Pacific Research Laboratories, Vashon, USA) with their apex exposed by 3 mm. The non-metal dummy implants served as a control to examine whether the presence of titanium implants, characterized by high stiffness and thermal conductivity,contributes to thermal change when cutting the bone model. The layered bone model consists of a cortical bone layer (density: 1.64 g/cc, thickness: 2 mm) and a cancellous bone layer (density: 0.32 g/cc, thickness: 8 mm). Bone cutting was performed using a piezoelectric surgery device (Variosurg3®, NSK Corporation, Tochigi, Japan). During bone cutting, the temperature at the implant apex was measured using a contact thermometer (TSU-0125 K-type thermometer, Tokai Hit Co., Ltd., Shizuoka, Japan) every 10 s (Fig. 1A). Cutting was performed at positions of 0 mm (adjacent to the implant), 1 mm, and 2 mm away from the implant, with a cutting depth of 3 mm (Fig. 1B). A specific cutting tip (H-SG8, NSK Corporation), set at the surgical mode and 100% power, was used. The cutting pressure was adjusted in advance using an electronic balance. One trained dentist performed all the cuts, and cutting was performed at a pressure of under almost 100–150 g [13, 20]. The cutting modes tested were continuous cutting and intermittent cutting. For intermittent cutting, cutting and resting alternated every 10, 5, or 3 s. Continuous irrigation was maintained throughout the process at a flow rate of 75 mL/min to prevent heat accumulation. Each cutting condition lasted 90 s excluding the resting time, and each experiment was repeated 5 times. The maximum temperature change was determined by subtracting the initial temperature from the peak temperature recorded during the experiments. All experiments were performed in a thermal chamber set to 37 °C.

### Histological analysis

Bone block from fresh porcine mandibular ramus were used to evaluate bone damage. The gingiva was carefully removed, and titanium implants were placed (diameter: 4.0 mm, length: 13 mm; Brånemark System® MkⅣ, Nobel Biocare). Bone around the implants was cut using a piezoelectric handpiece (Variosurg3® ultrasonic device, NSK Corporation) with an H-SG8 tip (NSK Corporation) under the surgical mode at 100% power. A cutting pressure of 100–150 g was applied. Cutting was performed at a distance of 0 mm from the implant and a cutting depth of 3 mm. Both continuous and intermittent cutting modes (5- and 3 s intervals) were tested. After bone cutting, the implants were removed, the mandibles were fixed in 4% paraformaldehyde for 24 h, dehydrated through an ethanol series, and decalcified in Plank-Rychlo solution for two weeks. The samples were embedded in paraffin and cut into 5 μm-thick sections, de-waxed, hydrated. Then, hematoxylin and eosin (HE) and Masson’s trichrome staining were performed. Four samples were prepared for each group. The specimens were examined under a digital microscope. Thermal injury, such as protein denaturation, the loss or cracking of the laminated structure, and carbonization, were identified, based on a previous study [21].

### Statistical analysis

The sample size for the bone model experiments was calculated using G-power (Heinrich Heine University of Dusseldorf, Germany) based on the results of a pilot study. An effect size (Cohen's D) was calculated, and a statistical power of 80% was set to determine the group size. For reliable statistical significance at α = 0.05, a total of 5 samples per group was required. The collected data are shown as mean ± standard deviation. Data analysis was performed using Bell Curve for Excel (Social Survey Research Information Co., Ltd., Tokyo, Japan). The normality of the data distribution was evaluated using the Shapiro–Wilk test. The significance of differences between two groups and multiple groups that were distributed normally was determined using a Student's t test and One-way ANOVA post hoc analyses using Tukey's HSD, respectively. Statistical significance was set at α = 0.05.

## Results

### Temperature increase during cutting of the bone model with titanium versus non-metal dummy implants (cutting at 0 mm from the implant)

**Fig. 2:**
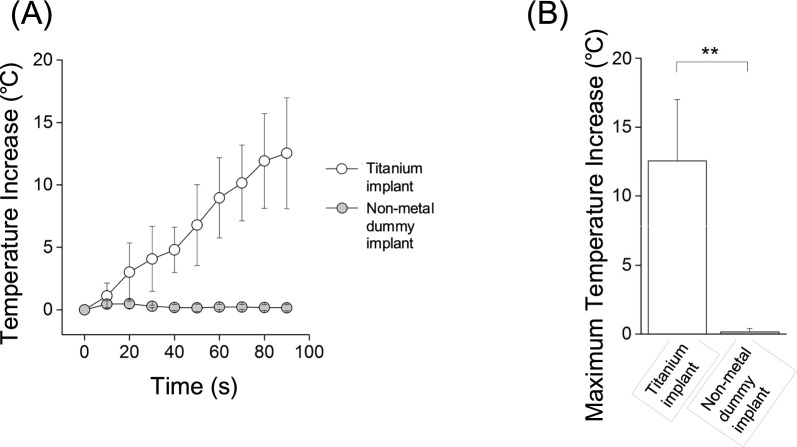
The temperature increase when cutting around titanium or non-metal dummy implants in the bone model experiemnts. Cutting 0 mm from the implant. **A** The time-course data of temperature changes when cutting. **B** Maximum temperature increase when cutting around titanium and non-metal dummy implants. n = 5 in each group. **p < 0.01

Almost no temperature increase was observed when cutting around the non-metal dummy implants, while the temperature increased when cutting around the titanium implants (Fig. 2A). The maximum temperature increase was significantly higher when cutting around titanium implants than the non-metal dummy implants (*p* < 0.01) (Fig. 2B).

### Temperature increase during cutting of the bone model with titanium implants (comparison by the distance from the implant)

**Fig. 3:**
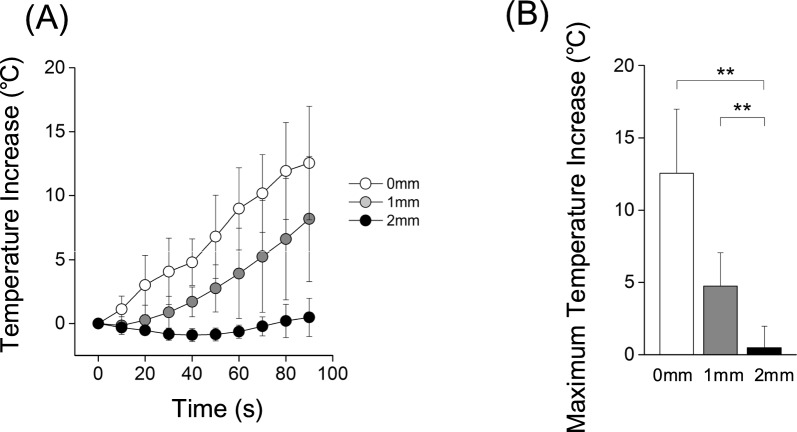
Effect of cutting distance (0, 1, and 2 mm) from the implant on the maximum temperature increase. The change in temperature over time and the maximum temperature increase at each distance are shown. Note that the data in cutting distance 0 mm is the same as the data of the titanium implant in Fig. 1. n = 5 in each group. **p < 0.01

When the bone model was cut 2 mm away from the implant, almost no temperature increase was observed. In contrast, the temperature increased when bone cutting occurred 1 mm or 0 mm away from the implants (Fig. 3A). The maximum temperature increase at 0, 1, 2 mm from the implants was 12.5 ± 4.5 °C, 4.7 ± 2.3 °C, and 0.5 ± 1.5 °C, respectively (Fig. 3B).

### Temperature increase during cutting of the bone model with titanium implants (comparison between continuous and intermittent cutting at 0 mm from the implant)

**Fig. 4:**
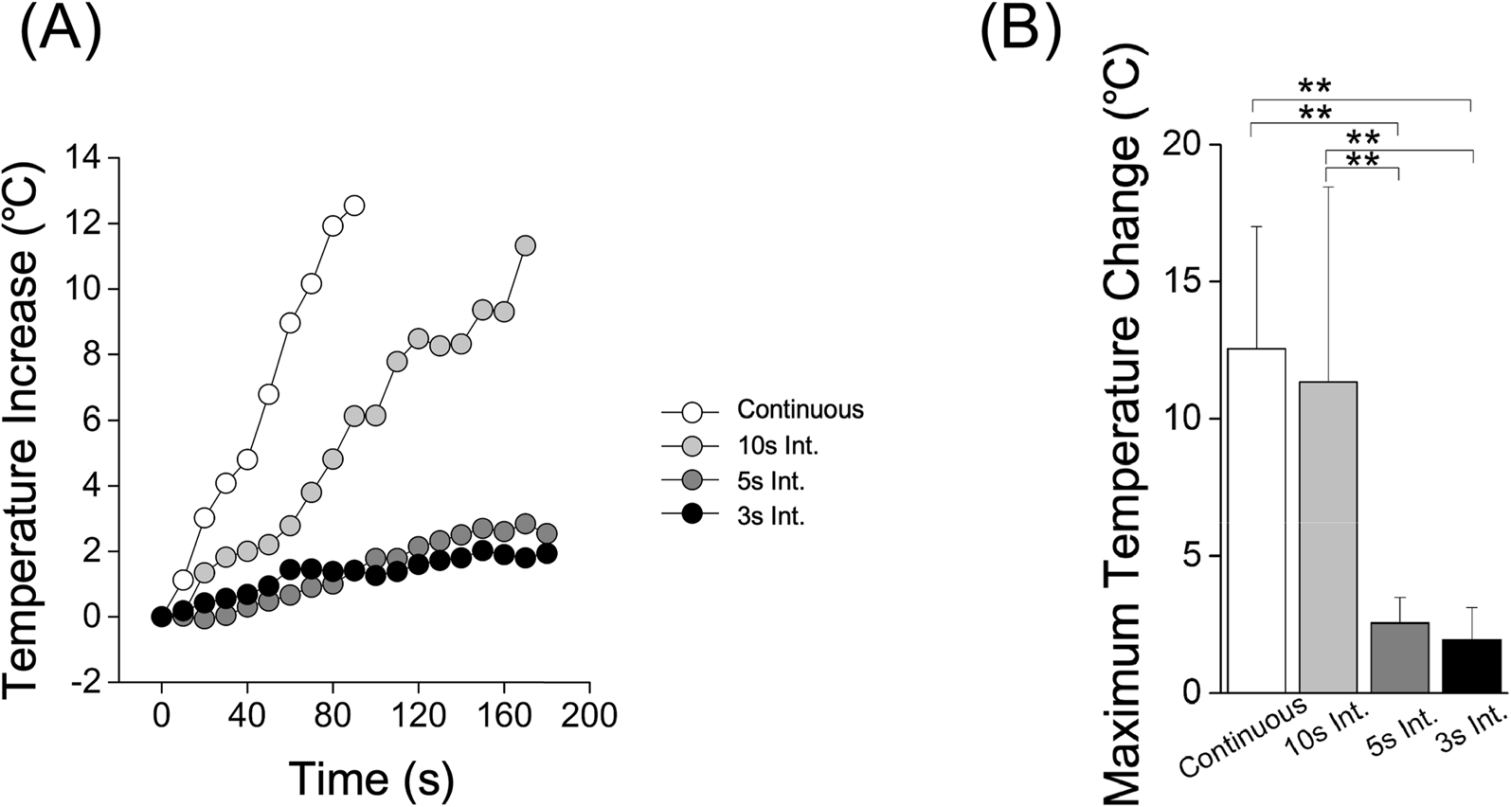
Maximum temperature increase under continuous and intermittent cutting conditions (10-, 5-, and 3 s intervals). The time-course data of temperature changes and the maximum temperature increase for each condition are shown. Note that the data in continuous cutting is the same as the data of the titanium implant in Fig. 1. Int.: Intermittent. n = 5 in each group. **p < 0.01

A temperature increase was observed in all groups. The increase was significantly larger during continuous and 10 s intermittent cutting (Fig. 4A). The maximum temperature increase during continuous and the 10 s intermittent cutting was 12.5 ± 4.5 °C and 11.3 ± 7.1 °C, respectively (Fig. 4B). Conversely, the maximum temperature increase during 5 s and 3 s intermittent cutting was 2.5 ± 1.0 °C and 1.9 ± 1.2 °C, respectively (Fig. 4B).

### Bone damage in porcine mandibles

**Fig. 5:**
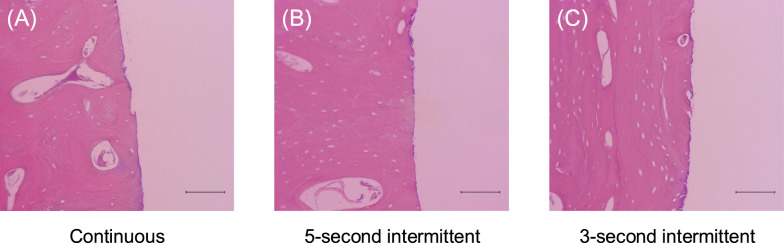
**A** Representative HE-stained porcine mandible specimen in the continuous cutting group. **B** Representative HE-stained porcine mandible specimen in the 5 s intermittent cutting group. **C** Representative HE-stained porcine mandible specimen in the 3 s intermittent cutting group. Scale bars = 100 μm

**Fig. 6:**
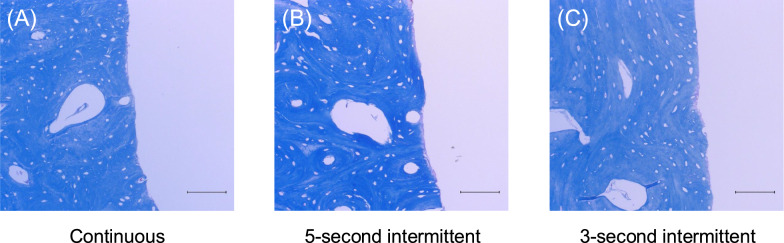
**A** Representative Masson’s trichrome-stained porcine mandible specimen in the continuous cutting group. **B** Representative Masson’s trichrome-stained porcine mandible specimen in the 5 s intermittent cutting group. **C** Representative Masson’s trichrome-stained porcine mandible specimen in the 3 s intermittent cutting group. Scale bars = 100 μm

Bone damage was evaluated using HE and Masson’s trichrome stained images. Representive HE stained images of each group are shown in Fig. 5A–C. Signs of thermal injury, such as protein denaturation, the loss or cracking of the laminated structure, and carbonization, were not observed in any of the groups, and no differences were noted among the groups. Representive Masson’s trichrome stained images of each group are shown in Fig. 6A–C. No findings consistent with protein denaturation in any of the groups, and no differences were noted among the groups.

## Discussion

In this study, a significantly higher temperature increase was observed in titanium implants compared to non-metal dummy implants fabricated from composite resin. This result is consistent with the findings of another study that investigated the use of ultrasonic tips to remove metal files for root canal treatment [22]. The mechanism of heat generation from piezoelectric surgery involves thermal conversion of vibration energy [14]. Metal is much harder than bone. When cutting close to metal, the cutting efficiency of piezoelectric surgery is significantly reduced as the vibration energy is not easily converted to facilitate cutting, leading to increased heat generation. Titanium and composite resin do not differ only in hardness but also in thermal conduction. In particular, composite resin has poor thermal conduction relative to titanium. In this study, temperature was measured at the implant apex. The result showed almost no temperature increase in the resin dummy implant, which may also be due to the lower thermal conductivity.

This study also demonstrated that the temperature increase during piezoelectric bone cutting varied significantly with the distance between the implant and the cutting position, suggesting that the conversion of vibration energy to heat may be greater when the cutting site is close to the implant. Importantly, the temperature increase effect becomes negligible when bone cutting is performed 2 mm away from the implant. However, keeping a 2 mm distance from the implant may be difficult clinically, resulting in significant alveolar bone loss. Conversely, bone loss can be reduced by removing bone closer to the implant.

To address this, we investigated whether temperature increase can be suppressed by intermittent cutting even when bone cutting is performed adjacent to the implant. Our results showed that continuous and 10 s intermittent cutting led to large temperature increases, whereas intermittent cutting at 3 s and 5 s intervals limited the temperature increase. Our results concur with the findings of a previous study on implant site preparation using piezoelectric surgery, which indicated that 4 s of continuous cutting led to a smaller temperature increase than 6 s of continuous cutting [13]. The short-intermittent cuts may allow time for cooling between heat accumulation and improve irrigation access to the narrow cutting area [23], leading to less increase in temperature.

Previous studies have consistently identified 47 °C as a critical temperature threshold for thermal bone damage based on the original work of Eriksson and Albrektsson in 1983 [24]. This threshold has since been corroborated by subsequent in vivo studies and systematic reviews, including the work of Kniha et al. [25]. In this study, the maximum temperatures in the continuous or 10 s intermittent cutting adjacent to the implants showed large temperature increases that approached the critical value, highlighting the potential risk of bone damage. In contrast, intermittent cutting at shorter intervals effectively maintained the temperatures well below this threshold. While previous studies have shown that intermittent cutting of bone for 10 s results in a small increase in temperature [26], in our study, 10 s intermittent cutting resulted in a large increase in temperature. This difference may be due to the presence of metal (a titanium implant) close to the cutting site, highlighting that continuous cutting times may need to be shorter than usual when cutting near an implant. These findings underscore the clinical relevance of using short-interval intermittent cutting to minimize the risk of bone necrosis during implant removal. By ensuring that the local temperature remains below the critical level, surgeons can help preserve bone viability [27] and thus, promote successful reimplantation in the same site.

In the present study, none of the samples demonstrated thermal injury. This supports our observation that short-interval intermittent cutting in the bone model experiments limited temperature increase. Even with the higher temperature increase (close to the critical threshold for thermal injury) during continuous cutting in the bone model experiments [24], histological evaluation did not reveal any thermal injury. This may be due to temperature being measured at the apex of the implant in this study, so the bone did not experience the large temperature increase that leads to thermal injury. Of note, our results do not suggest that thermal injury absolutely will not occur even when bone cutting is performed adjacent to the implant with piezoelectric surgery.

In HE stained bone specimens following thermal injury, several characteristic histological features can be observed: empty lacunae within the bone matrix, the bone matrix may show increased basophilia in the bone matrix, reflecting protein denaturation and coagulation necrosis, and a semi-annular or band-like necrotic zone at the site of thermal injury [28]. In this study, we did not use live animals, but instead used the jawbone of a pig that was already dead, so we were able to evaluate protein denaturation but not coagulation necrosis and empty lacunae. The detection of empty lacunae is one of the most characteristic features of bone thermal injury, indicateing the loss of osteocytes due to thermal necrosis [29]. Although it was possible to observe osteocytes and empty lacunae in the HE stained images in this study, the animals were already dead when subjected to piezoelectric surgery. Therefore, the cellular responses may differ from those in living animals, and it cannot be excluded that some osteocytes had already died prior to bone cutting by piezoelectric surgery. For this reason, osteocyte viability was not included in the results of this study. In the future, studies in living animals may provide further insights.

Despite the promising results, this study has several limitations. Bone model and bone block in this study lacked soft-tissue coverage and body fluid perfusion. Therefore, the absolute temperature values reported here should not be directly applied to clinical conditions. There are heat dissipation paths other than bone, such as irrigation water and blood around the implant neck, however, there are almost no dissipation paths except for the surrounding bone near the implant apex, and the heat can be harmful to the around bone. Therefore, this study focused on temperature changes at the implant apex. Although direct assessment of temperature propagation into the surrounding bone at the implant apex could provide more relevant information, which was technically challenging. By evaluating the temperature of the surrounding bone, the effect of heat on the bone may be estimated with greater accuracy. Although the HE and Masson’s trichrome-stained images showed no apparent thermal injury, these conventional stains are known to have low sensitivity for detecting subtle protein denaturation. Therefore, further studies using more sensitive techniques are warranted to clarify microscopic thermal alterations. Additionally, the effects of power and the choice of tip used in piezoelectric surgery, the amount and temperature of irrigation, and cutting pressure were not evaluated. Future studies should investigate the effects of additional variables, such as cutting pressure and irrigation flow rate, on temperature changes to identify optimal parameters for safe and effective bone cutting around implants using piezoelectric surgery.

## Conclusions

This study demonstrated that piezoelectric surgery did not result in bone damage. However, continuous bone cutting adjacent to the implants generated heat. While, short-interval intermittent cutting effectively minimize heat generation. These findings can be used to establish a safe and effective method for implant removal using piezoelectric surgery.

## Data Availability

The data that supports the findings of this study is available from the corresponding author upon reasonable request.
